# 4-[5-(4-Fluoro­phen­yl)-1*H*-imidazol-4-yl]pyridine

**DOI:** 10.1107/S1600536809005650

**Published:** 2009-02-21

**Authors:** Pierre Koch, Dieter Schollmeyer, Stefan Laufer

**Affiliations:** a Institute of Pharmacy, Department of Pharmaceutical and Medicinal Chemistry, Eberhard-Karls-University Tübingen, Auf der Morgenstelle 8, 72076 Tübingen, Germany; b Department of Organic Chemistry, Johannes Gutenberg-University Mainz, Duesbergweg 10-14, 55099 Mainz, Germany

## Abstract

In the title compound, C_14_H_10_FN_3_, the imidazole ring makes dihedral angles of 28.2 (1) and 36.60 (9)° with the pyridine ring and the 4-fluoro­phenyl ring, respectively. The pyridine ring forms a dihedral angle of 44.68 (9)° with the 4-fluoro­phenyl ring. Inter­molecular N—H⋯N hydrogen bonds are observed in the crystal structure.

## Related literature

For the biological activity of the title compound, see: Liverton *et al.* (1999 ▶). For applications of functionalized 5(4)-(4-fluoro­phen­yl)-4(5)-(pyridin-4-yl)imidazoles, see: Koch *et al.* (2008 ▶), Peifer *et al.* (2006 ▶).
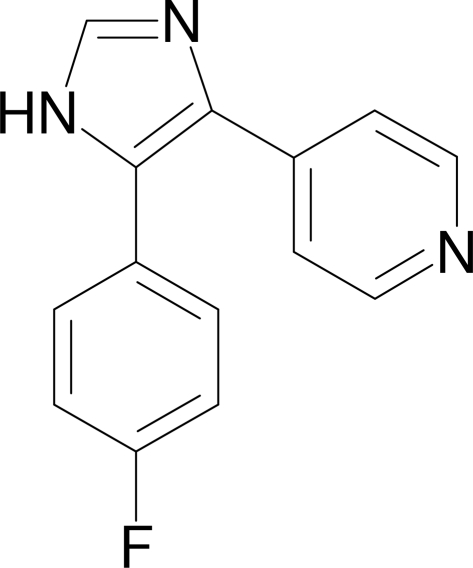


## Experimental

### 

#### Crystal data

C_14_H_10_FN_3_
*M* *_r_* = 239.25
Orthorhombic, 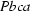

*a* = 9.217 (2) Å
*b* = 8.1064 (5) Å
*c* = 30.665 (5) Å
*V* = 2291.1 (6) Å^3^
*Z* = 8
Cu *K*α radiation
μ = 0.80 mm^−1^
*T* = 193 K
0.54 × 0.20 × 0.13 mm

#### Data collection

Enraf–Nonius CAD-4 diffractometer
Absorption correction: none
2121 measured reflections
2121 independent reflections
1707 reflections with *I* > 2σ(*I*)
3 standard reflections frequency: 60 min intensity decay: 2%

#### Refinement

*R*[*F* ^2^ > 2σ(*F* ^2^)] = 0.076
*wR*(*F* ^2^) = 0.201
*S* = 1.09
2121 reflections
163 parameters
H-atom parameters constrained
Δρ_max_ = 0.58 e Å^−3^
Δρ_min_ = −0.54 e Å^−3^

### 

Data collection: *CAD-4 Software* (Enraf–Nonius, 1989 ▶); cell refinement: *CAD-4 Software*; data reduction: *CORINC* (Dräger & Gattow, 1971 ▶); program(s) used to solve structure: *SIR97* (Altomare *et al.*, 1999 ▶); program(s) used to refine structure: *SHELXL97* (Sheldrick, 2008 ▶); molecular graphics: *PLATON* (Spek, 2009 ▶); software used to prepare material for publication: *PLATON*.

## Supplementary Material

Crystal structure: contains datablocks I, global. DOI: 10.1107/S1600536809005650/im2099sup1.cif

Structure factors: contains datablocks I. DOI: 10.1107/S1600536809005650/im2099Isup2.hkl

Additional supplementary materials:  crystallographic information; 3D view; checkCIF report

## Figures and Tables

**Table 1:** Hydrogen-bond geometry (Å, °)

*D*—H⋯*A*	*D*—H	H⋯*A*	*D*⋯*A*	*D*—H⋯*A*
N1—H1⋯N15^i^	0.89	1.94	2.815 (3)	164

Symmetry code: (i) 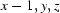.

**Fig. 1.**
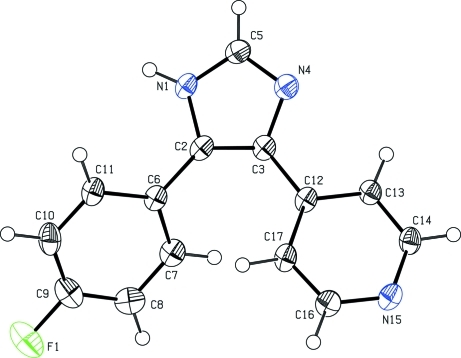
View of compound I. Displacement ellipsoids are drawn at the 50% probability level. H atoms are depicted as circles of arbitrary size.

**Fig. 2.**
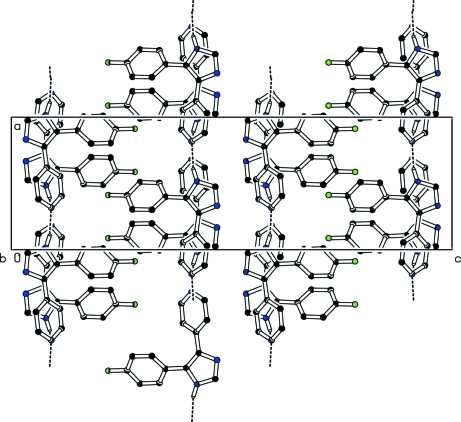
Part of the crystal packing of compound I. The hydrogen bonds are represented by dashed lines. View along b axis.

## supplementary crystallographic information

### Comment

5(4)-(4-Fluorophenyl)-4(5)-(pyridin-4-yl)imidazole derivatives with various substitution patterns have been considered to be potential p38 MAP kinase inhibitors (Liverton *et al.* 1999, Koch *et al.* 2008, Peifer *et al.* 2006).

The molecular structure of compound I is shown in Figure 1. The imidazole ring realises dihedral angles of 28.2 (1)° and 36.60 (9)° with the pyridine ring and the 4-fluorophenyl ring, respectively. The pyridine ring encloses a dihedral angle of 44.68 (9)° with the 4-fluorophenyl ring.

The crystal packing (Figure 2) shows N1—H1 of the imidazole ring to form an intermolecular N–H···N hydrogen bond towards pyridine (N15) resulting in a infinite chain parallel to the *a* axis. The hydrogen bond measures 1.94 Å.

### Experimental

1-(4-Fluorophenyl)-2-(pyridin-4-yl)ethane-1,2-dione (46 mg, 0.2 mmol), formaldehyde (15 µ*L*, 0.2 mmol, 37% aq. solution), ammonium acetate (154 mg, 2.0 mmol) and 1 ml glacial acetic acid were combined in a reaction vial. The reaction vessel was heated in a CEM microwave reactor for 5 min at 453 K (initial power 200 W), after which a stream of compressed air cooled the reaction vessel. The reaction mixture was added dropwise to a concentrated NH_4_OH solution at 0 °C. The formed colorless precipitate was collected by filtration, washed with water and dried (yield: 43 mg, 90%). Crystals of compound I suitable for X-ray diffraction were obtained by slow evaporation at 298 K of a solution of n-hexane - diethyl ether (3:2).

### Refinement

Hydrogen atoms attached to carbons were placed at calculated positions with C—H = 0.95 Å (aromatic C-atoms). The position of H1 was determined from the difference Fourier map. All H atoms were refined in the riding-model approximation with isotropic displacement parameters (set at 1.2 times of the *U*_eq_ of the parent atom).

### Crystal data

**Table:**

C_14_H_10_FN_3_	*D*_x_ = 1.387 Mg m^−^^3^
*M**_r_* = 239.25	Melting point: 285.5 K
Orthorhombic, *P**b**c**a*	Cu *K*α radiation, λ = 1.54178 Å
Hall symbol: -P 2ac 2ab	Cell parameters from 25 reflections
*a* = 9.217 (2) Å	θ = 31–53°
*b* = 8.1064 (5) Å	µ = 0.80 mm^−^^1^
*c* = 30.665 (5) Å	*T* = 193 K
*V* = 2291.1 (6) Å^3^	Needle, colourless
*Z* = 8	0.54 × 0.20 × 0.13 mm
*F*(000) = 992	

### Data collection

**Table:**

Enraf–Nonius CAD-4 diffractometer	*R*_int_ = 0.0000
Radiation source: rotating anode	θ_max_ = 69.6°, θ_min_ = 2.9°
graphite	*h* = 0→11
ω/2θ scans	*k* = 0→9
2121 measured reflections	*l* = −36→0
2121 independent reflections	3 standard reflections every 60 min
1707 reflections with *I* > 2σ(*I*)	intensity decay: 2%

### Refinement

**Table:**

Refinement on *F*^2^	Primary atom site location: structure-invariant direct methods
Least-squares matrix: full	Secondary atom site location: difference Fourier map
*R*[*F*^2^ > 2σ(*F*^2^)] = 0.076	Hydrogen site location: inferred from neighbouring sites
*wR*(*F*^2^) = 0.201	H-atom parameters constrained
*S* = 1.09	*w* = 1/[σ^2^(*F*_o_^2^) + (0.1422*P*)^2^ + 0.0554*P*] where *P* = (*F*_o_^2^ + 2*F*_c_^2^)/3
2121 reflections	(Δ/σ)_max_ < 0.001
163 parameters	Δρ_max_ = 0.58 e Å^−^^3^
0 restraints	Δρ_min_ = −0.54 e Å^−^^3^

### Special details

**Table:**

Geometry. All e.s.d.'s (except the e.s.d. in the dihedral angle between two l.s. planes) are estimated using the full covariance matrix. The cell e.s.d.'s are taken into account individually in the estimation of e.s.d.'s in distances, angles and torsion angles; correlations between e.s.d.'s in cell parameters are only used when they are defined by crystal symmetry. An approximate (isotropic) treatment of cell e.s.d.'s is used for estimating e.s.d.'s involving l.s. planes.
Refinement. Refinement of *F*^2^ against ALL reflections. The weighted *R*-factor *wR* and goodness of fit *S* are based on *F*^2^, conventional *R*-factors *R* are based on *F*, with *F* set to zero for negative *F*^2^. The threshold expression of *F*^2^ > σ(*F*^2^) is used only for calculating *R*-factors(gt) *etc*. and is not relevant to the choice of reflections for refinement. *R*-factors based on *F*^2^ are statistically about twice as large as those based on *F*, and *R*- factors based on ALL data will be even larger.

### Fractional atomic coordinates and isotropic or equivalent isotropic displacement parameters (Å^2^)

**Table:**

	*x*	*y*	*z*	*U*_iso_*/*U*_eq_	
F1	0.0873 (2)	0.0708 (3)	0.21998 (6)	0.0596 (6)	
N1	−0.0150 (2)	0.2988 (2)	0.42177 (7)	0.0243 (5)	
H1	−0.1070	0.2883	0.4130	0.029*	
C2	0.1085 (2)	0.2755 (3)	0.39720 (8)	0.0224 (5)	
C3	0.2212 (3)	0.3123 (3)	0.42585 (8)	0.0227 (5)	
N4	0.1672 (2)	0.3595 (3)	0.46674 (7)	0.0285 (5)	
C5	0.0257 (3)	0.3479 (3)	0.46279 (9)	0.0279 (6)	
H5	−0.0408	0.3708	0.4857	0.033*	
C6	0.1015 (2)	0.2250 (3)	0.35030 (8)	0.0228 (5)	
C7	0.1984 (3)	0.2872 (3)	0.31898 (9)	0.0286 (6)	
H7	0.2682	0.3669	0.3277	0.034*	
C8	0.1955 (3)	0.2353 (3)	0.27519 (9)	0.0338 (6)	
H8	0.2639	0.2764	0.2547	0.041*	
C9	0.0907 (3)	0.1233 (3)	0.26276 (9)	0.0365 (7)	
C10	−0.0096 (3)	0.0625 (3)	0.29215 (9)	0.0353 (6)	
H10	−0.0819	−0.0131	0.2828	0.042*	
C11	−0.0043 (3)	0.1128 (3)	0.33599 (8)	0.0274 (6)	
H11	−0.0731	0.0705	0.3562	0.033*	
C12	0.3785 (3)	0.3036 (3)	0.41935 (8)	0.0216 (5)	
C13	0.4700 (3)	0.4057 (3)	0.44398 (8)	0.0246 (5)	
H13	0.4299	0.4814	0.4643	0.030*	
C14	0.6182 (3)	0.3961 (3)	0.43855 (8)	0.0282 (6)	
H14	0.6783	0.4650	0.4559	0.034*	
N15	0.6824 (2)	0.2927 (3)	0.40951 (7)	0.0287 (5)	
C16	0.5937 (3)	0.1937 (3)	0.38606 (9)	0.0290 (6)	
H16	0.6367	0.1193	0.3659	0.035*	
C17	0.4456 (3)	0.1942 (3)	0.38966 (8)	0.0264 (6)	
H17	0.3885	0.1218	0.3724	0.032*	

### Atomic displacement parameters (Å^2^)

**Table:**

	*U*^11^	*U*^22^	*U*^33^	*U*^12^	*U*^13^	*U*^23^
F1	0.0846 (15)	0.0588 (13)	0.0354 (10)	−0.0016 (11)	−0.0071 (9)	−0.0100 (9)
N1	0.0210 (10)	0.0106 (9)	0.0414 (12)	−0.0012 (7)	0.0000 (8)	−0.0015 (8)
C2	0.0225 (11)	0.0050 (10)	0.0398 (14)	0.0003 (7)	0.0019 (9)	0.0031 (9)
C3	0.0277 (13)	0.0048 (10)	0.0355 (12)	−0.0005 (8)	0.0005 (9)	0.0007 (8)
N4	0.0293 (11)	0.0182 (10)	0.0379 (12)	−0.0021 (8)	0.0020 (9)	−0.0029 (8)
C5	0.0273 (12)	0.0166 (11)	0.0399 (14)	−0.0008 (9)	0.0058 (10)	−0.0027 (10)
C6	0.0234 (11)	0.0078 (10)	0.0371 (13)	0.0037 (8)	−0.0022 (9)	0.0011 (9)
C7	0.0308 (12)	0.0139 (11)	0.0410 (15)	0.0016 (9)	−0.0012 (10)	0.0027 (10)
C8	0.0379 (14)	0.0257 (13)	0.0377 (15)	0.0053 (10)	0.0035 (11)	0.0069 (11)
C9	0.0499 (17)	0.0278 (14)	0.0319 (14)	0.0083 (11)	−0.0085 (12)	−0.0012 (11)
C10	0.0383 (14)	0.0215 (12)	0.0460 (16)	−0.0033 (11)	−0.0113 (12)	−0.0041 (11)
C11	0.0277 (12)	0.0139 (11)	0.0407 (14)	−0.0017 (9)	−0.0044 (10)	0.0020 (9)
C12	0.0239 (12)	0.0070 (10)	0.0337 (13)	−0.0007 (8)	−0.0014 (9)	0.0044 (8)
C13	0.0282 (12)	0.0151 (11)	0.0306 (12)	−0.0020 (9)	−0.0004 (9)	−0.0006 (9)
C14	0.0294 (13)	0.0194 (12)	0.0357 (13)	−0.0036 (9)	−0.0039 (10)	−0.0016 (10)
N15	0.0231 (10)	0.0223 (11)	0.0408 (12)	0.0008 (8)	−0.0016 (9)	0.0022 (9)
C16	0.0286 (13)	0.0156 (12)	0.0429 (15)	0.0050 (9)	−0.0006 (10)	−0.0024 (10)
C17	0.0273 (12)	0.0091 (10)	0.0427 (15)	−0.0001 (9)	−0.0044 (10)	−0.0017 (9)

### Geometric parameters (Å, °)

**Table:**

F1—C9	1.380 (3)	C8—H8	0.9500
N1—C5	1.372 (3)	C9—C10	1.382 (4)
N1—C2	1.378 (3)	C10—C11	1.406 (4)
N1—H1	0.8936	C10—H10	0.9500
C2—C3	1.393 (3)	C11—H11	0.9500
C2—C6	1.496 (3)	C12—C13	1.402 (3)
C3—N4	1.402 (3)	C12—C17	1.414 (3)
C3—C12	1.465 (3)	C13—C14	1.379 (3)
N4—C5	1.313 (3)	C13—H13	0.9500
C5—H5	0.9500	C14—N15	1.359 (3)
C6—C11	1.403 (3)	C14—H14	0.9500
C6—C7	1.405 (3)	N15—C16	1.353 (3)
C7—C8	1.407 (4)	C16—C17	1.370 (3)
C7—H7	0.9500	C16—H16	0.9500
C8—C9	1.379 (4)	C17—H17	0.9500
			
C5—N1—C2	108.4 (2)	F1—C9—C10	119.7 (3)
C5—N1—H1	124.3	C9—C10—C11	119.8 (2)
C2—N1—H1	127.3	C9—C10—H10	120.1
N1—C2—C3	104.0 (2)	C11—C10—H10	120.1
N1—C2—C6	121.9 (2)	C6—C11—C10	120.7 (2)
C3—C2—C6	134.2 (2)	C6—C11—H11	119.6
C2—C3—N4	111.0 (2)	C10—C11—H11	119.6
C2—C3—C12	129.9 (2)	C13—C12—C17	117.0 (2)
N4—C3—C12	119.0 (2)	C13—C12—C3	119.6 (2)
C5—N4—C3	104.5 (2)	C17—C12—C3	123.4 (2)
N4—C5—N1	112.1 (2)	C14—C13—C12	119.8 (2)
N4—C5—H5	123.9	C14—C13—H13	120.1
N1—C5—H5	123.9	C12—C13—H13	120.1
C11—C6—C7	117.4 (2)	N15—C14—C13	123.1 (2)
C11—C6—C2	120.5 (2)	N15—C14—H14	118.5
C7—C6—C2	122.1 (2)	C13—C14—H14	118.5
C6—C7—C8	122.2 (2)	C16—N15—C14	116.8 (2)
C6—C7—H7	118.9	N15—C16—C17	123.9 (2)
C8—C7—H7	118.9	N15—C16—H16	118.0
C9—C8—C7	118.3 (3)	C17—C16—H16	118.0
C9—C8—H8	120.9	C16—C17—C12	119.3 (2)
C7—C8—H8	120.9	C16—C17—H17	120.3
C8—C9—F1	118.8 (3)	C12—C17—H17	120.3
C8—C9—C10	121.5 (3)		
			
C5—N1—C2—C3	0.3 (2)	C7—C8—C9—C10	0.0 (4)
C5—N1—C2—C6	−178.6 (2)	C8—C9—C10—C11	−1.1 (4)
N1—C2—C3—N4	−0.9 (2)	F1—C9—C10—C11	178.5 (2)
C6—C2—C3—N4	177.8 (2)	C7—C6—C11—C10	1.6 (3)
N1—C2—C3—C12	177.2 (2)	C2—C6—C11—C10	−178.8 (2)
C6—C2—C3—C12	−4.1 (4)	C9—C10—C11—C6	0.2 (4)
C2—C3—N4—C5	1.2 (2)	C2—C3—C12—C13	153.7 (2)
C12—C3—N4—C5	−177.14 (19)	N4—C3—C12—C13	−28.4 (3)
C3—N4—C5—N1	−1.0 (3)	C2—C3—C12—C17	−27.5 (4)
C2—N1—C5—N4	0.4 (3)	N4—C3—C12—C17	150.5 (2)
N1—C2—C6—C11	−37.4 (3)	C17—C12—C13—C14	0.0 (3)
C3—C2—C6—C11	144.1 (2)	C3—C12—C13—C14	178.9 (2)
N1—C2—C6—C7	142.3 (2)	C12—C13—C14—N15	1.1 (4)
C3—C2—C6—C7	−36.3 (4)	C13—C14—N15—C16	−1.5 (4)
C11—C6—C7—C8	−2.7 (3)	C14—N15—C16—C17	0.9 (4)
C2—C6—C7—C8	177.6 (2)	N15—C16—C17—C12	0.1 (4)
C6—C7—C8—C9	2.0 (4)	C13—C12—C17—C16	−0.5 (3)
C7—C8—C9—F1	−179.6 (2)	C3—C12—C17—C16	−179.4 (2)

### Hydrogen-bond geometry (Å, °)

**Table:**

*D*—H···*A*	*D*—H	H···*A*	*D*···*A*	*D*—H···*A*
N1—H1···N15^i^	0.89	1.94	2.815 (3)	164

Symmetry codes: (i) *x*−1, *y*, *z*.
